# Labeling for Big Data in radiation oncology: The Radiation Oncology Structures ontology

**DOI:** 10.1371/journal.pone.0191263

**Published:** 2018-01-19

**Authors:** Jean-Emmanuel Bibault, Eric Zapletal, Bastien Rance, Philippe Giraud, Anita Burgun

**Affiliations:** 1 Radiation Oncology Department, Georges Pompidou European Hospital, Assistance Publique – Hôpitaux de Paris (AP-HP), Paris Descartes University, Paris Sorbonne Cité, Paris, France; 2 INSERM UMR 1138 Team 22: Information Sciences to support Personalized Medicine, Paris Descartes University, Sorbonne Paris Cité, Paris, France; 3 Biomedical Informatics and Public Health Department, Georges Pompidou European Hospital, Assistance Publique – Hôpitaux de Paris (AP-HP), Paris Descartes University, Paris Sorbonne Cité, Paris, France; ENEA Centro Ricerche Casaccia, ITALY

## Abstract

**Purpose:**

Leveraging Electronic Health Records (EHR) and Oncology Information Systems (OIS) has great potential to generate hypotheses for cancer treatment, since they directly provide medical data on a large scale. In order to gather a significant amount of patients with a high level of clinical details, multicenter studies are necessary. A challenge in creating high quality Big Data studies involving several treatment centers is the lack of semantic interoperability between data sources. We present the ontology we developed to address this issue.

**Methods:**

Radiation Oncology anatomical and target volumes were categorized in anatomical and treatment planning classes. International delineation guidelines specific to radiation oncology were used for lymph nodes areas and target volumes. Hierarchical classes were created to generate The Radiation Oncology Structures (ROS) Ontology. The ROS was then applied to the data from our institution.

**Results:**

Four hundred and seventeen classes were created with a maximum of 14 children classes (average = 5). The ontology was then converted into a Web Ontology Language (.owl) format and made available online on Bioportal and GitHub under an Apache 2.0 License. We extracted all structures delineated in our department since the opening in 2001. 20,758 structures were exported from our “record-and-verify” system, demonstrating a significant heterogeneity within a single center. All structures were matched to the ROS ontology before integration into our clinical data warehouse (CDW).

**Conclusion:**

In this study we describe a new ontology, specific to radiation oncology, that reports all anatomical and treatment planning structures that can be delineated. This ontology will be used to integrate dosimetric data in the Assistance Publique—Hôpitaux de Paris CDW that stores data from 6.5 million patients (as of February 2017).

## Introduction

The increasing number of parameters that need to be taken into account to achieve precision medicine makes it almost impossible to design dedicated trials [1]. Hypothesis-generating studies, using Big Data approaches can help in identifying relevant prognostic or predictive factors to be explored later in randomized controlled trials, in order to generate level-I evidence. Such studies need a high number of patients and features for meaningful analysis, using for example machine learning algorithms. This calls for the participation of several centers for patient and treatment data pooling and integration. In the field of radiation oncology, medical data is already highly structured, through the use of Oncology Information and “Record-and-Verify” systems. Data can be easily extracted with the precise features of treatment planning (dosimetry) and delivery. However, this data can have very heterogeneous labels that will need time-consuming curation and unification. It is particularly true for anatomical and target volumes labeling.

Using routine radiation oncology data requires respecting a set of principles, to make it more accessible. These principles, known as the FAIR Data Principles [2], initially developed for research data, are now being extended to clinical trials and routine care data. Medical data are stored in totally secure environments, and within such confined the data must be Findable, Accessible, Interoperable, and Reusable for research purposes Behind FAIR principles is the notion that algorithms may be used to search for relevant data, to analyze the data sets, and to mine the data for knowledge discovery. Electronic health record data cannot be fully shared but efforts can be made to make vocabularies and algorithms reusable and enable multi-site collaborations. To achieve that goal, the radiation oncology community must pave the road for semantic frameworks that the sources and the users could agree upon in the future. Besides usual quantitative data (dose, etc), standard representation of anatomical regions and target volumes is required to study, for example, radiation complications. In this article, we present methods to annotate radiation data with harmonized vocabularies and to integrate radiation data with clinical and genomic data in translational platforms.

### Background

Large Hospital Information Systems (CERNA, EPIC) have no functionality specific to radiation oncology. There are currently several domain-specific softwares for radiation oncology planning: MOSAIQ (Elekta, Stockholm, Sweden), ARIA (Varian, Palo Alto, California, USA), Multiplan and Tomotherapy Data Management System (Accuray, Sunnyvale, California, USA) and iPlan (Brainlab, Munich, Germany). Each of these treatment planning and record-and-verify systems has its own anatomical structures labelling, which is not consistent across platforms, making it difficult to extract and analyze dosimetric data on a multicenter large scale. Using knowledge management with concept recognition, classification and mapping would lead to an accurate ontology that could be used to unify data in clinical data warehouses, thus facilitating data reuse and study replication in cancer centers. Previous efforts have included the creation of precise naming nomenclatures [3], without defining classes or concepts. The American Association of Physicists in Medicine also setup a task group for Standardizing Nomenclature for Radiation Therapy (AAPM TG 263) and will publish their recommendations soon [4].

### Ontologies of anatomical structures

In order to be able to use and share information, we need a controlled common vocabulary, a list of terms that have been enumerated explicitly. These terms must be hierarchically collected in a taxonomy. In addition to these parent-child associations, networks between concepts can be developed to create a thesaurus. Beyond these, an ontology is a formal naming and definition of the types, properties, and interrelationships of the concepts that exist in a domain. They organize in a formal logical format, standardized terms that are both human-readable and machine-processable. Most of them are based on description logics to ensure consistency. They are distributed as open source components of information systems that can be maintained separately from software and therefore shared among many different users and applications. The use of ontology is already widespread in biomedical domains outside of radiation oncology. They have also been recognized as a necessary tool in the basic sciences, e.g., the Gene Ontology provides the foundation for annotating genes.

The Foundational Model of Anatomy (FMA) was developed by the University of Washington to serve as an ontology of anatomical structures that could be used for multiple purposes [5]. It has been used as a basic ontology in several projects developed by the W3C including the NeuroImaging Model. However, it was created as a reference ontology for anatomical entities and is not adapted to represent anatomical volumes and delineation features specific to radiation oncology. Drawing similar conclusions for medical imaging, the radiology community has developed RadLex an application ontology that incorporates and accommodates all salient anatomical knowledge necessary to manage anatomical information related to radiology [6]. RadLex was used, for example, to annotate positron emission tomography-computed tomography (PET-CT) images and support studies on these semantically enriched data [7]. These terminologies can be organized in repositories, such as the Bioportal or the UMLS Metathesaurus [8]. However, no ontology did include specific radiation oncology terms, which led to the creation of the Radiation Oncology Ontology (ROO) by Dekker et al [9], that reused other ontologies and added specific radiation oncology terms such as Region Of Interest (ROI), Target Volumes (GTV, CTV, PTV), Dose-Volume Histograms (DVH). Still, the ROO does not provide concepts for all anatomical or target volume currently used in most radiation oncology department and has not been updated since July 2015. For example, lymph nodes levels, that are essential for the planning of nodal CTV in radiotherapy, are not included [10,11]. We chose to create anontology dedicated to radiation oncology structures with a high level of detail that aligned to reference ontologies like FMA.

#### i2b2: A platform for clinical data warehousing (CDW)

The Georges Pompidou European Hospital (HEGP) is an 800-bed academic hospital located in southwest Paris belonging to Assistance Publique-Hôpitaux de Paris (AP-HP), with focus on oncology and cardiovascular diseases. In 2012, the French National Cancer Institute (INCa) granted eight SIRICs (Site de Recherche Intégré sur le Cancer in French, or Integrated Cancer Research Site) labels in France. SIRICs’ ambitions are to provide new operational resources to oncology research, to optimize and accelerate the production of knowledge and to favor knowledge dissemination and application in patient care. The CARPEM (CAncer Research and PErsonalized Medicine) program is one of these eight SIRICs and HEGP is strongly involved in CARPEM. Data from 750,000 patients are stored in the HEGP clinical data warehouse, including 14,000 cancer patients treated with radiation. The HEGP clinical data warehouse (CDW) relies on the Informatics for Integrating Biology and the Bedside (i2b2) model—an open source infrastructure developed by Harvard Medical School and adopted by more than 130 academic hospitals around the world [12,13]. The i2b2 warehouse uses an Entity-Attribute-Value (EAV) data model for its adaptability and dynamic nature. Concepts are stored separately in a hierarchical data model. We designed the Radiation Oncology Structures ontology to unify data extracted from our Record-and-Verify System (ARIA) before integrating the data into our i2b2 CDW [14].

## Methods

### Ontology design

To design an ontology is to create and organize a set of concepts (or classes) in a given domain. An application ontology is an ontology designed for a specific use or application focus and whose scope is specified through testable use cases. Application ontologies often re-use reference canonical ontologies such as the FMA to construct ontological classes and relationships between classes. We designed the Radiation Oncology Structures (ROS) ontology to fit the specific needs of radiotherapy, while being aligned to existing ontologies, such as the Foundational Model of Anatomy (FMA). For example, a class of head and neck anatomical structures represents all organs at risk in the head and neck area. Head and neck lymph nodes (LN) levels are instances of this class. The head and neck LN level II is an instance of the class of head and neck LN. A class can have subclasses that represent concepts that are more specific than the superclass. We can divide the class head and neck LN level II in IIa and IIb subclasses. Alternatively, we can divide a class of head and neck LN level by laterality, right and left, or we can divide ideally by laterality and level. We chose this model for the ROS ontology, because it gives the highest granularity (or level of details) that we will need for easy and straight dosimetric analysis on a large scale. In practical terms, developing an ontology requires defining classes and arranging the classes in a taxonomic (subclass–superclass) hierarchy. Several ontology design approaches are possible. We adopted a bottom up approach to identify and cluster the terms into concepts, then a top-down development process to organize the concepts and design the ontology.

We started with extracting structure names from our Record-and-Verify System with VARIAN Application Programming Interface (ESAPI) [15] (ARIA, Varian, Palo Alto, California, United States) and cluster them into the most general concepts in the domain (anatomical vs target volumes) and subsequent specialization of the concepts. For each subclasses, lymph nodes levels were named according to international radiation oncology delineation guidelines [10,16–19]. Because the numbering of these levels might overlap between areas, each level’s name includes the superclass it belongs to. When all concepts were defined, we used Protégé 5.2.0, a free, open-source editor and framework to build the ontology [20,21]. Each class is linked to existing FMA [22] or SNOMED Clinical Terms [23] concepts.

### Data integration into the clinical data warehouse (CDW)

We evaluated the coverage of our ontology by using it to annotate all HEGP radiation oncology data with ROS concepts and integrate them into the HEGP clinical data warehouse. This study was approved by the IRB and ethics committee CPP Ile-de-France II. IRB Committee # 00001072. Study reference # CDW_2015_0024. Data on treatment planning and delivery in our institution (2001–2016) were extracted from the ARIA system using reverse engineering and the VARIAN ESAPI [15] for dose-volume histograms (DVH). Structures labels were sorted and filtered by number of occurrences. Each of them was then matched to the ROS ontology before integration into the CDW.

## Results

### The ROS ontology

We created four hundred and seventeen classes, with a maximum number of subclasses of 14 and an average number of 5. The first three superclasses are Anatomical Volumes, Target Volumes and Miscellaneous Volumes. For the anatomical volumes, we created seven subclasses: head, neck, thorax, abdomen, pelvis, limb and spine. For each of the anatomical classes, we created subclasses for lymph nodes, vessels, vertebrae or bones, and specific organs. For each paired organ, we specified left or right with a class. For target volumes, we used Biological Target Volumes (BTV), Clinical Target Volumes (CTV), Gross Tumor Volumes (GTV), Internal Target Volumes (ITV) and Planning Target Volumes (PTV). Within each of these subclasses, we created a primary target volume that includes the gross primary and tumor volume and a nodal target volume that includes all involved nodal regions. For the Clinical Target Volumes, subclasses were created for each risk level (low, intermediary and high). The Miscellaneous Volumes class includes an applicator volume (for brachytherapy), an external volume and a pacemaker volume.

We mapped the ROS to the Unified Medical Language System (UMLS) using the UMLS terminology service Application Programming Interface (UTS API). The mapping was realized in four steps: first, we tried searching ROS terms using an exact match strategy (case insensitive, but whitespace sensitive). Then, we normalized the unmatched terms using the normalization function provided by the UMLS and attempt to match them against similarly normalized terms in the UMLS. In some cases, the UMLS normalization is too conservative and fails to identify an existing concept of the UMLS. We extended the normalization in two additional steps: (1) we removed numbers at the end of terms. For example, the term “Cervical Vertebrae 01” is normalized to “Cervical Vertebrae” and performed a normalized search in the UMLS. (2) We extended the normalization by removing also words related to the laterality (i.e. “left” and “right”). For example, “Left Rib 03” is normalized to “Rib,” and matched against normalized terms from the UMLS. The first two strategies identified equivalent concepts, whereas the extended normalization identified broader concepts. Overall, we were able to find a mapped concept in the UMLS for 81% of the ROS classes (S1 Table). The remaining unmapped classes are constituted of the lymph node areas and the target volumes (S2 Table).

**Fig 1:**
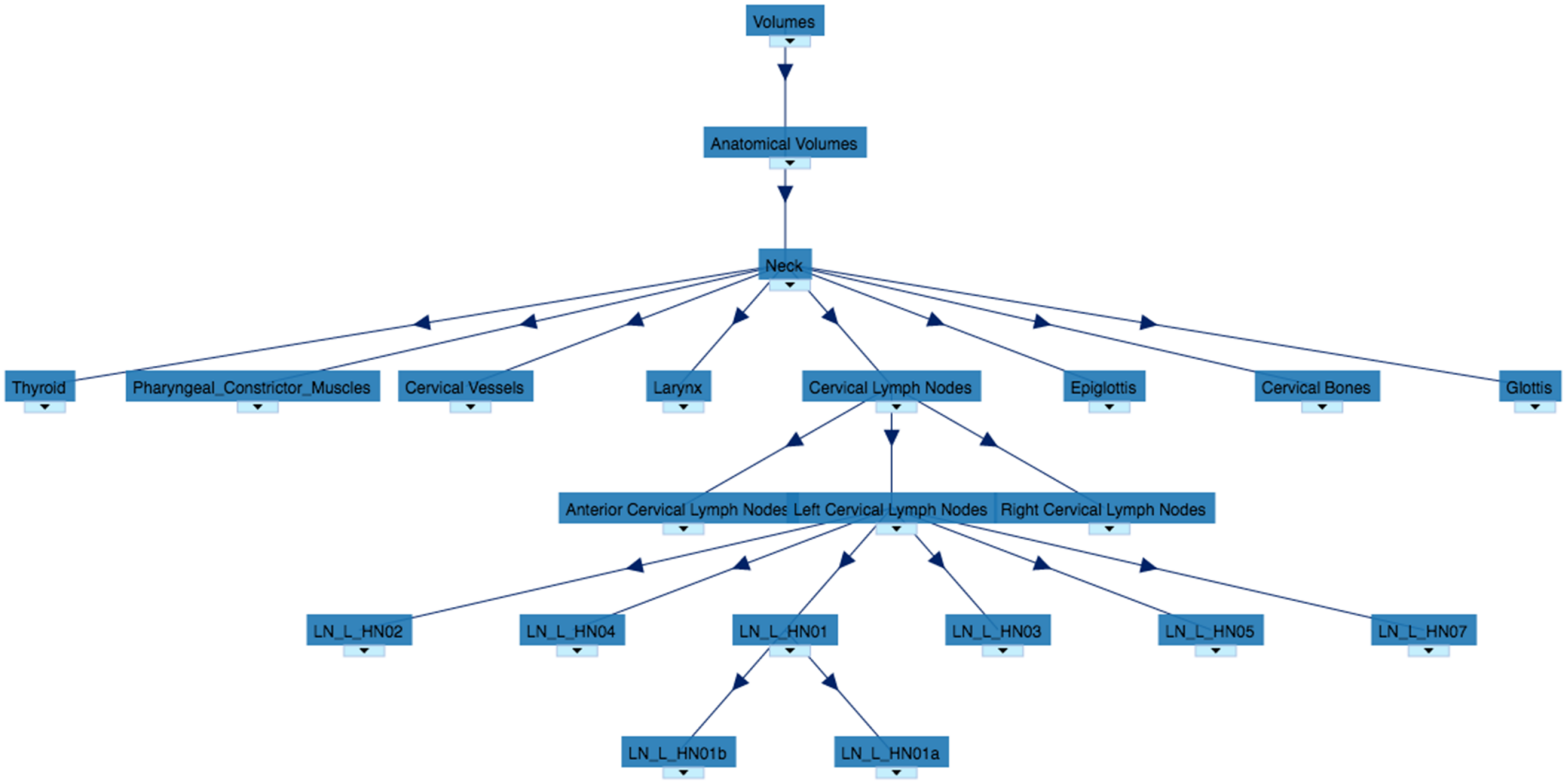
Map of the ROS ontology from the first superclass to the cervical lymph nodes area I class.

The ontology is available online on Bioportal (Fig 1) [24] and GitHub[25] in.owl,.csv and.rdf formats.

### Use case from HEGP

**Fig 2:**
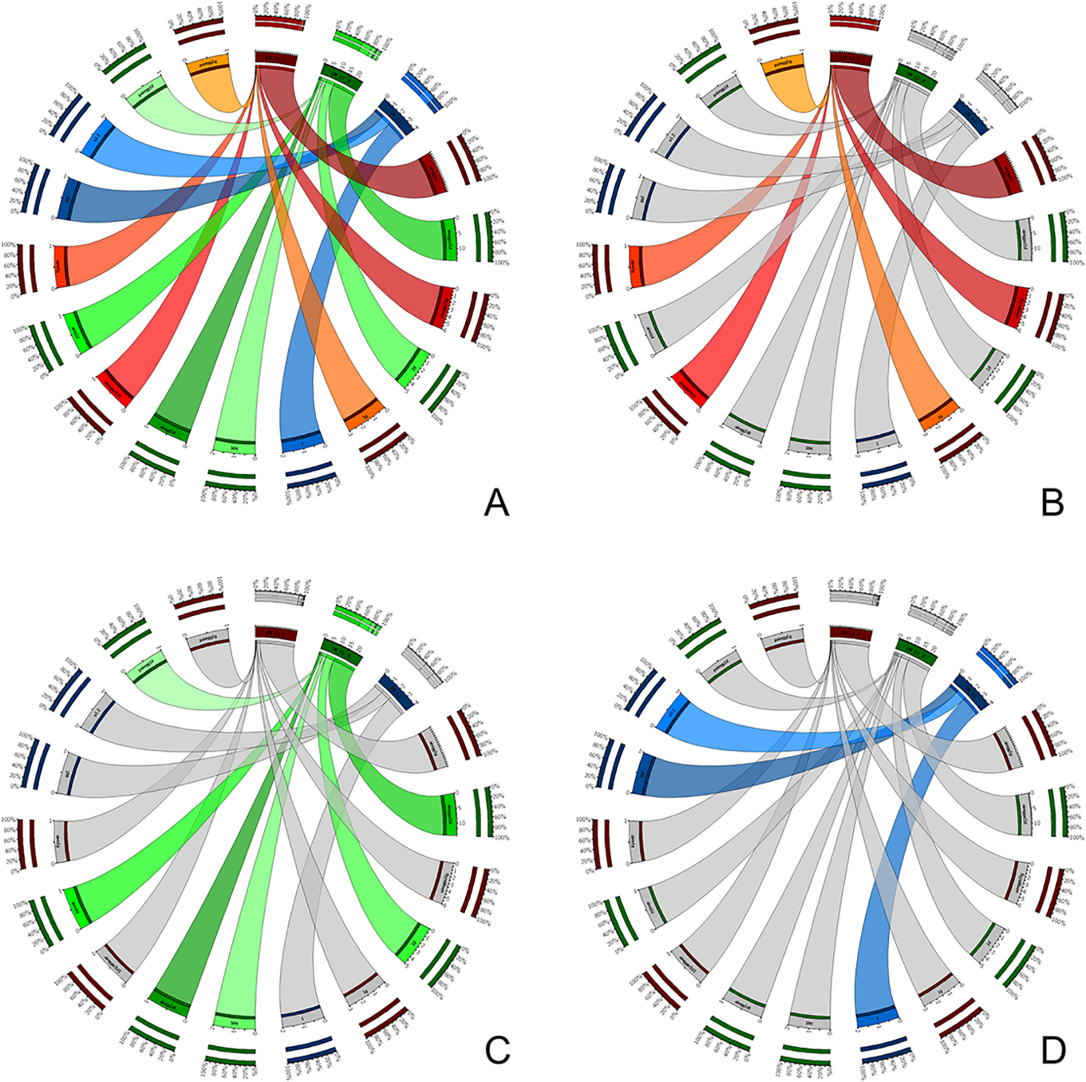
Visualization of heterogeneity reduction for cervical lymph nodes area I. A: all area I labels. B: Label LN_C1_L (red). C: Label LN_C1_R (green). D: Label LN_C1 (blue).

**Fig 3:**
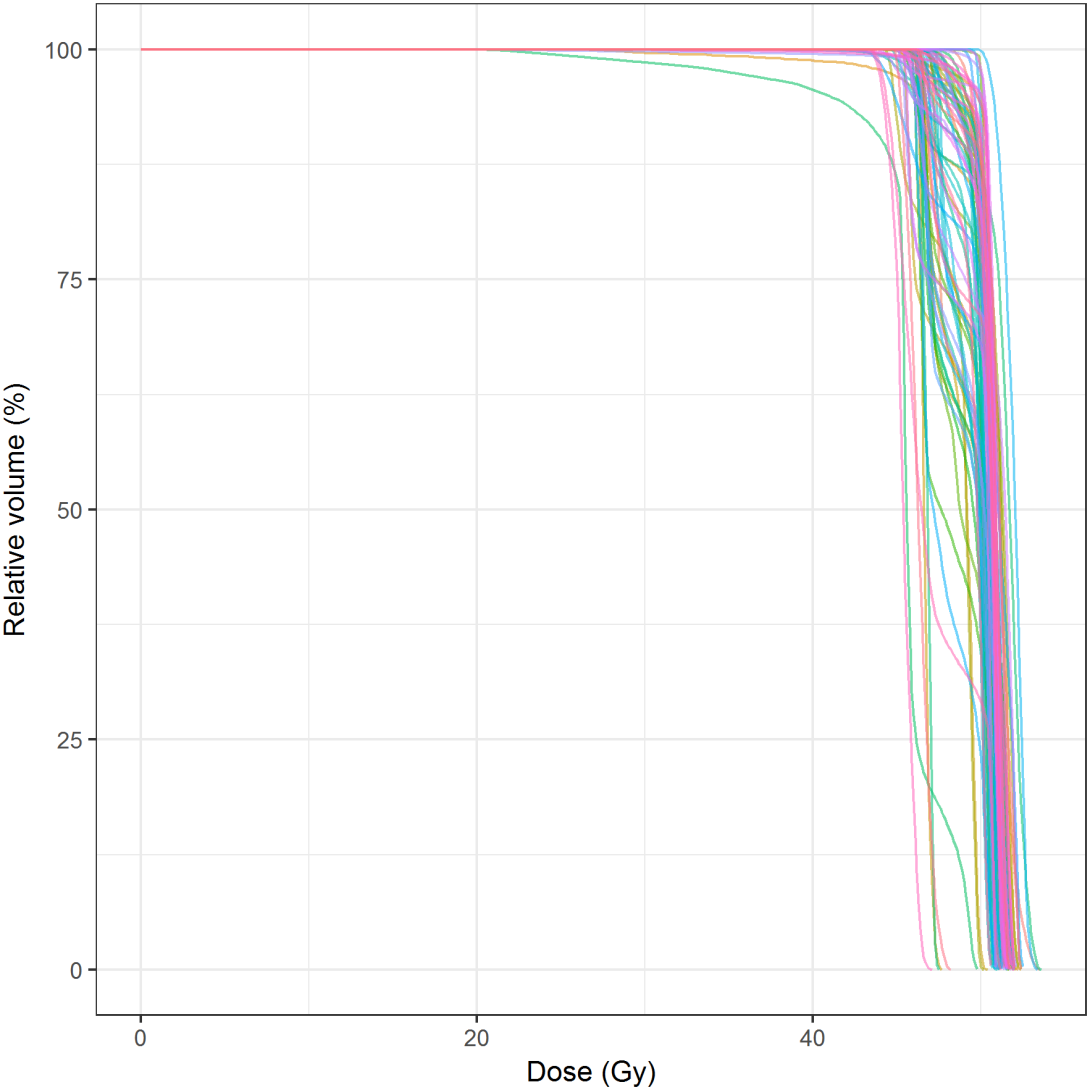
Dose Volume Histogram for 84 patients treated with neo-adjuvant chemoradiation for rectal cancer generated from the dosimetric data extracted from the treatment planning system and integrated into our i2b2 CDW.

With the aim of integrating data from our Treatment Planning and Record-and-Verify System (ARIA, Varian, Palo Alto, California, United States) into our institutional CDW, we extracted the following dosimetric data: Course ID, Plan Setup ID, Reference Point ID, Total Dose Limit, Daily Dose Limit, Session Dose Limit. For each treatment fraction we included Date/Time, duration of the activity and text comment. We also needed to include Dose-Volume Histograms (DVH) for each treatment. We ensured that structures labeling was consistent across the population. A total of 20,758 different structures labels were created for treatment planning between 2001 (opening of our department) and 2017. We applied the ROS ontology to our data to correct inconsistencies in spelling, laterality, or lymph node levels before integration. Structure names were clustered to create classes (Fig 2): LN_C1_L (Left Cervical Lymph node Level 1) in red, LN_C1_R (Right Cervical Lymph node Level 1) in green and LN_C1 (All Cervical Lymph node Level 1) in For this area, we reduced the labels from 15 to 3 classes from the ROS ontology (contributions from each initial labels are shown in red, green and blue ribbons, which respectively contribute to the ROS labels LN_C1_L, LN_C1_R and LN_C1). As a demonstration, we integrated all the data from a cohort of 262 patients using the ROS Ontology. We reconstructed the Dose Volume Histogram for the mesorectum, directly from the data stored in the i2b2 CDW for a subgroup of 84 patients treated with neoadjuvant chemoradiation for rectal cancer (Fig 3).

## Discussion

### Why did we need a specific ontology?

Several anatomical ontologies are already available, and it would be easier to re-use existing ontologies. But no existing ontology matches the classifications we use on a daily basis for delineating lymph node areas. We needed to reuse our data from our CDW, and since all our labels have been created according to lymph node areas guidelines, we decided to create a new ontology for that purpose. The ROS ontology describes commonly contoured (anatomical and treatment planning) structures for radiation treatment planning. We extracted 20,758 structures labels (created over a 16 years period in our radiation department), classified and categorized them to produce the ontology. Lymph nodes delineation international guidelines are provided. This ontology was created to standardize the integration of radiation oncology data into clinical data warehouses for multicenter studies. The high granularity of this ontology will allow precise dosimetric evaluations.

A key benefit of biomedical ontologies is sharing and reuse: We have validated the coverage and the consistency of the ROS ontology by using it to annotate the radiation oncology data of 14,000 patients treated at HEGP radiation oncology department between 2001 and 2016. The ontology is now made available to be used in other hospitals. The ROS ontology can be downloaded from BioPortal and re-used in other centers and applications [24]. Ontologies are separate from application code that uses them. This separation of ontologies from applications permits both the commercial and public sectors to cooperate in developing and using these ontologies for the benefit of the entire scientific community. We believe that similar benefits will accrue for the ontology that we have developed. In fact, ontologies enable to create many data intensive applications and therefore could become a critical technology for "big data" radiation oncology. Any institution is able to use our ontology by matching their existing data, which is fairly easy, since we only used standard concepts that rely on international guidelines.

### Benefits of Big Data for radiation oncology

Radiation oncology generates a large amount of data for each patient. This data, unlike in most medical specialties, are well structured because it is already stored in digital systems and can be extracted. A comprehensive Electronic Health Record for any cancer patient will be around 8 GB, with genomic and imaging data being the largest contributors. Creating a predictive model in radiation oncology requires a significant variety and heterogeneity of the data types that need to be included. This represents in itself a significant challenge. This large amount of high-quality data, can be leveraged with Big Data approaches, such as machine learning, in order to create predictive models [26–29]. Previously published studies have described the methods that could be used, on a smaller scale [30] or for international data-sharing [31,32]. Here we present the first actual implementation of our approach in a clinical data warehouse. The end-goal will be to create a Learning Health System that will guide the physician to personalize treatment, according to the predicted efficacy or toxicity [33]. Patients that will have a good treatment-response could benefit from de-escalated dose for example [34].

### Possible improvements and perspectives

The ROS ontology can be used by anyone because it is available online under an Apache 2 Licence. Improvements could include new target volumes that could appear in the future. A higher level of details could also be added, if there were an actual need for it (*i*.*e*. only if these structures are indeed delineated in routine). The granularity of our ontology is not an obstacle to its implementation in other departments (which is underway in 4 others APHP radiation therapy centers), since the level of granularity is adapted to the level of the treating center (thanks to the hierarchical structure of the ontology). For example, if a center does not delineate each cervical lymph-node levels (1 through X for example), the superclass above can be used (simply use the left or right cervical lymph nodes class). However, we must stress that using detailed level to delineate and create target volumes is standard-of-care, since an international consensus is now available [10].

The ROS ontology will be used to harmonize structures labels generated by the five radiation oncology departments from AP-HP, the largest public health service in Europe, with 39 hospitals. Five radiation oncology departments treat each year around 8,000 patients (122,000 fractions). AP-HP has a CDW that includes data from 6.5 million patients as of February 2017. Following the successful example of HEGP, data from all AP-HP radiation oncology departments will be integrated within the central AP-HP CDW.

## Conclusion

Because we needed to harmonize structures labels before we could integrate treatment planning and delivery into our CDW, we created an ontology specific to radiation oncology delineation. Four hundred and seventeen classes were created with a maximum of 14 children classes (average = 5). The ROS ontology allowed us to reduce the 20758 structures created during 15 years in our institution to only 417 classes that matched across any patients or treatments.

## Supporting information

S1 Table Supp file 1—RO_UMLSMappings.xls. Classes of the ROS ontology mapped to the UMLS.
(XLSX)

S2 Table Supp file 2—Supp file 2—RO-unmapped_UMLSMappings.xls. Classes of the ROS ontology unmapped in the UMLS.
(XLSX)
